# Designing malaria vaccines to circumvent antigen variability^✩^

**DOI:** 10.1016/j.vaccine.2015.09.110

**Published:** 2015-11-01

**Authors:** Amed Ouattara, Alyssa E. Barry, Sheetij Dutta, Edmond J. Remarque, James G. Beeson, Christopher V. Plowe

**Affiliations:** a Institute for Global Health and Howard Hughes Medical Institute, University of Maryland School of Medicine, Baltimore, MD, USA; b Malaria Research and Training Center, University of Sciences, Techniques and Technology, Bamako, Mali; c Division of Population Health and Immunity, Walter and Eliza Hall Institute for Medical Research, Parkville, Australia; d Department of Medical Biology, University of Melbourne, Melbourne, Australia; e Walter Reed Army Institute of Research, Silver Spring, MD, USA; f The Biomedical Primate Research Center, Rijswijk, Netherlands; g Burnet Institute, Melbourne, Victoria, Australia; h Department of Microbiology, Monash University, Victoria, Australia

**Keywords:** Malaria, Vaccine, Diversity, Heterologous, Allele-specific efficacy, Cross-protection

## Abstract

Prospects for malaria eradication will be greatly enhanced by an effective vaccine, but parasite genetic diversity poses a major impediment to malaria vaccine efficacy. In recent pre-clinical and field trials, vaccines based on polymorphic *Plasmodium falciparum* antigens have shown efficacy only against homologous strains, raising the specter of allele-specific immunity such as that which plagues vaccines against influenza and HIV. The most advanced malaria vaccine, RTS,S, targets relatively conserved epitopes on the *P. falciparum* circumsporozoite protein. After more than 40 years of development and testing, RTS,S, has shown significant but modest efficacy against clinical malaria in phase 2 and 3 trials. Ongoing phase 2 studies of an irradiated sporozoite vaccine will ascertain whether the full protection against homologous experimental malaria challenge conferred by high doses of a whole organism vaccine can provide protection against diverse strains in the field. Here we review and evaluate approaches being taken to design broadly cross-protective malaria vaccines.

## 1. Introduction

A malaria vaccine that prevents infection would reduce malaria morbidity and mortality and accelerate malaria eradication efforts [1]. Leading approaches for achieving this goal include subunit vaccines that target sporozoites and blood stage parasites and that block transmission of gametocytes to mosquitoes, as well as whole organism vaccines intended to block sporozoite invasion of liver cells, entirely preventing blood stage infection and gametocytogenesis [1,2].

The most advanced malaria vaccine, RTS,S/AS01, targets *Plasmodium falciparum* circumsporozoite protein (CSP; expressed on pre-erythrocytic stages) and moderately reduces the risk of clinical malaria (efficacy 40–70% in different populations) but does not entirely prevent infection in field trials [3]. Subunit blood stage vaccines have also had limited success in reducing clinical malaria or parasitemia [4–7]. One such blood stage vaccine candidate, FMP2.1/AS01, based on the *P. falciparum* apical membrane antigen 1 (AMA1), had no overall efficacy against clinical malaria. However, the FMP2.1/AS01 vaccine had significant allele-specific efficacy against clinical malaria caused by parasites identical to the vaccine strain with respect to specific amino acid positions previously identified as important determinants of allele-specific natural immunity *in vitro* [8], in field trials [9] and in response to vaccination with a monovalent vaccine [7,10].

Immunization with “Combination B” a vaccine (Table 1) based on merozoite surface protein 2 (MSP2), MSP1 and ring-infected erythrocyte surface antigen (RESA) had overall efficacy in reducing parasitemia, but efficacy was restricted to infections with vaccine-like alleles of MSP2 [4]. Thus both blood stage vaccines, FMP2.1/AS01 and Combination B, selectively controlled the growth in the blood of parasites with vaccine-type variants of the target antigens (Table 1). These efficacy trials demonstrated that strategies are needed to overcome extensive [11], sometimes extreme [9], sequence diversity in vaccine antigen encoding genes [12].

Surveys of vaccine antigen diversity in natural parasite populations have revealed regions of proteins under balancing selection as immune targets and determined the frequency of different polymorphisms and haplotypes in different populations worldwide [13]. Meta-analyses combining all available data [9,14] and studies of laboratory isolates from many different countries [15] have been useful in cataloging global diversity and identifying the most common haplotypes circulating locally and worldwide. These studies have shown that antigen alleles can be sub-grouped on the basis of sequence similarity at codons for immunologically relevant amino acids into a manageable number of subgroups [14–16]. However, the link between these haplotype groupings and antigenic relatedness has not been well established [17,18].

The antigen haplotypes (“strains”) included in all malaria vaccines currently in development were chosen based on available laboratory isolates (“freezer epidemiology”), and in many cases have not taken into account haplotype prevalences at clinical trial sites or in intended target populations. This may explain the failure of some malaria vaccines tested to date [12]. Measuring the genetic and antigenic diversity of *P. falciparum* antigens may facilitate the design of broadly cross-protective multi-strain malaria vaccines that include relevant diverse alleles of antigens addressing all life cycle stages [16].

## 2. Impact of vaccination on genetic diversity of breakthrough infections

### 2.1. Vaccine-induced selection in other pathogens

Antigenic selection, which is the accumulation of amino acids changes that render an antigen less recognizable by the immune system, is seen during influenza virus seasonal monitoring. This surveillance has identified novel strains that can successfully infect both vaccinated and unvaccinated hosts and that are therefore incorporated into the following year's vaccine [19]. In other pathogens, selection may occur more slowly. For example, a 10-year follow-up of the Hib vaccine [20] and a 4-year genetic surveillance following the introduction of the meningococcal C vaccine in the United Kingdom [21] showed non-significant increases in genetic distance from the vaccine strain. These observations illustrate the capacity of microorganisms to adapt to modified host environments.

A sieve analysis consists of measuring the allele-specific immunological barrier to infection or disease [22]. Such a sieve analysis of human immunodeficiency virus-1 (HIV-1) sequences following immunization with the MRKAd5 vaccine showed a greater distance between HIV vaccine recipients sequences compared to those of the control group [23].

**Fig. 1:**
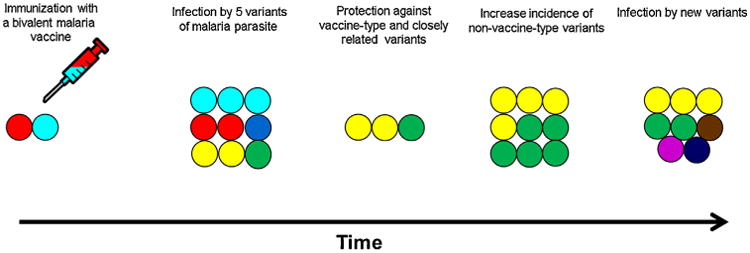
Effect of allele-specific efficacy on breakthrough infections. The malaria vaccine contains two variants (red and cyan) of a polymorphic antigen (A). When an individual is infected by five different parasites with five variants (cyan, red, dark blue, yellow and green) of the same proteins (B), malaria clinical episodes with homologous (red and cyan) and closely related variants (dark blue) are prevented (C). Heterologous variants (yellow and green) may increase within the individual and the whole population (D, E).

**Fig. 2:**
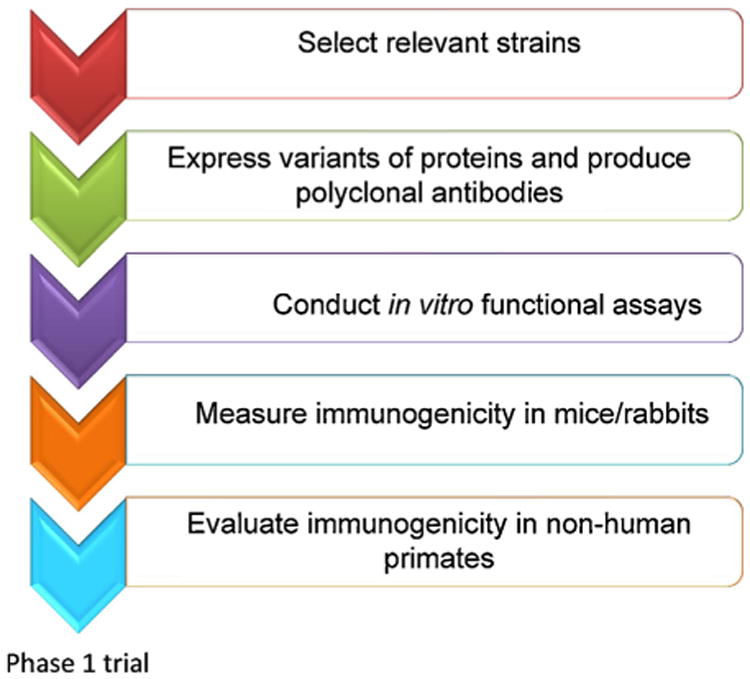
Screening procedure for the design of a broadly efficacious subunit malaria vaccine. The first step of the selection process consists of using field samples to assess the baseline genetic diversity of vaccine candidate antigens. Selected antigen candidates are then expressed and used to produce polyclonal antibodies. Following this step, *in vitro* functional assays are conducted followed by a clinical trial in non-human primate for successful candidates.

Vaccine-induced selection and/or antigenic drift (changes in gene frequencies due to chance) following immunization may reduce overall vaccine efficacy in the long term [24] (Fig. 1). To mitigate this risk, molecular epidemiology studies are needed to assess the baseline genetic diversity of vaccine candidate antigens (Fig. 2) and the diversity and distribution of alleles following immunization with polymorphic antigens. Moreover the antigenic variation of vaccine candidate antigens should be assessed very early in vaccine development (Fig. 2) to select alleles based on antigenic differences, and not just genetic differences, as these may not fully overlap [17,18].

### 2.2. Malaria vaccines

The *P. falciparum* genome consists of approximately 5400 genes distributed over 14 chromosomes [25]. Almost 4% of the genes encode proteins that are thought to be involved in immune evasion and host parasite interactions [26]. At least 31% of the predicted proteins have one or more transmembrane domains or GPI anchored proteins, including several of the surface antigens that have been tested in vaccine trials (*e.g*. AMA1, MSP2, CSP) [26]. These antigens are targets of humoral immunity, particularly during malaria infection [4]. In endemic areas, an individual acquires only partially protective immune responses following repeated infections from multiple strains of the parasite, suggesting that the immune response may be allele-specific to some extent. This allele specificity decreases with age, reflecting repeated exposure and the broadening of the immune response [27].

#### 2.2.1. Pre-erythrocytic subunit vaccines

Previous data on malaria vaccine-induced selection are limited by the small number of malaria vaccines that have reached phase 2 trials. RTS,S/AS01, directed against highly conserved B-cell epitopes in the repeat region of the pre-erythrocytic antigen CSP, is the vaccine candidate that has progressed the furthest in clinical testing [3]. Initial studies of the allele-specific efficacy of RTS,S in phase 2 trials measured the difference in the frequency of individual CSP alleles in vaccinees compared to controls. Haplotype analyses in these studies were restricted to Th2R and Th3R alleles encoding T-cell epitopes, and did not examine the repeat region thought to be the dominant target of protective antibodies [28,29], and no selection for parasites with non-vaccine strain CSP was detected [30] (Table 1). While small sample sizes could have resulted in missing relatively small selective effects in these initial studies, prospective molecular epidemiological studies also suggest that CSP is not the target of naturally-acquired allele-specific immunity directed at either the B-cell or T-cell epitopes [31]. However, strain-specific efficacy was seen in a much larger phase 3 trial of RTS,S in children at 11 African sites. RTS,S was better at protecting against malaria caused by vaccine-type parasites in older children, but not in the youngest infants [32]. In older children, but not in the youngest infants, various measures of efficacy were approximately 10 to 15 percentage points lower when they were evaluated against non–vaccine type parasites. This recent result supports the idea that careful selection of vaccine strains and diversity-covering approaches may help to improve the modest efficacy of this leading malaria vaccine [33].

#### 2.2.2. Whole organism vaccines

Another approach to overcome allele-specific immunity might be to overwhelm the parasite by targeting not just one or a few but thousands of antigens with a whole parasite vaccine. In a recent Controlled Human Malaria Infection trial, an irradiated sporozoite-based vaccine induced full protection in all 6 recipients of the highest dose administered [34]. This experimental challenge trial tested the PfSPZ Vaccine against homologous challenge with a parasite clone very nearly identical to the vaccine strain. The results of ongoing phase 2 studies in Mali and Tanzania will provide preliminary measures of the efficacy of this whole organism vaccine against heterologous strains.

#### 2.2.3. Blood-stage vaccines

The combination B malaria vaccine significantly reduced parasitemia overall, but increased parasitemia with non-vaccine type parasites (based on MSP2) in vaccinated individuals in a phase 2b trial in Papua New Guinea [4]. However, other vaccines based on MSP1 (YSLFQKEKMVL) and other peptides (SPf66) [35] had no effect on the allele frequencies of MSP1 sequences in vaccinees compared to controls (Table 1). No evidence of selection was observed, although some evidence of decreased multiplicity of infection (MOI) was observed in vaccinated participants indicating that the vaccine may not have an allele-specific efficacy effect. If antigen selection is taking place following immunization with a potential allele-specific vaccine, it may reduce the overall efficacy of the vaccine. Indeed, the efficacy of some vaccines against other pathogens that are based on polymorphic antigens has been attributed to the inclusion of diverse forms of the same antigen in the formulation [36]. Therefore, an understanding of allele-specific efficacy is an important step in the development of a multivalent broadly efficacious malaria vaccine.

## 3. Designing cross-protective malaria vaccines

Vaccines for other polymorphic pathogens have been designed to overcome genetic diversity by including diverse strains, *e.g*. inactivated trivalent polio vaccine [37], or multiple variants of vaccine antigens, *e.g*. multivalent pneumococcal vaccines [38]. As a first step toward a broadly efficacious malaria vaccine, the antigenic variants known to be most prevalent in the target populations, or, as in the case of HPV, the most virulent strains should be those selected to include in the vaccine. Malaria vaccines currently under development have not been designed in this way. Instead, in part because of a lack of molecular epidemiological information on haplotype prevalence, conveniently available laboratory isolates have been used to derive recombinant vaccine antigens. Recently, new approaches have been taken by researchers to cover the diversity of malaria vaccine candidates [39]. These approaches have been made possible by recent progress in the understanding of parasite biology, and advances in bioinformatics, genomics and the availability of the parasite genome [40,41].

### 3.1. Diversity covering approaches

#### 3.1.1. Immuno-dampening and bioinformatics approaches

Most decisions about which variant(s) of a polymorphic antigen to include in a broadly efficacious malaria vaccine have been arbitrary. An unlucky choice may result in a failed vaccine candidate, as likely occurred with the FMP1/AS02A MSP1 vaccine, which includes a variant of the antigen that turned out to be relatively rare in nature [6,11,42]. To optimize variant selection for vaccine design, researchers have used different approaches to reduce the number of strains to include in vaccine formulations. Rabbit immunization experiments using a mixture of two AMA1 variants (FVO and 3D7) yielded functional antibody responses equal to the homologous antigens at half the dose [43]. Responses to strains not covered by either component (*e.g*. M24), however, were not broadened [43]. Immunization with a mixture of three AMA1 alleles designed to cover amino acid diversity represented by 355 GenBank entries yielded a broad functional antibody responses to a small panel of laboratory strains [39]. The mechanism underlying this broadened response was elucidated by comparing the epitope range of rabbit polyclonal antibodies against a polyvalent and monovalent AMA1 vaccine [44]. It was found that in addition to responding to all variable epitopes, antibody responses following immunization with a mixture of four natural AMA1 variants were refocused toward conserved functional epitopes, a phenomenon termed epitope dilution. Stochastic modeling of affinity maturation using a two-epitope model of AMA1 further explained a mechanism for epitope dilution [45]. After identifying amino acids that were binding residues of inhibitory antibodies [8], seven out of ten residues in AMA1 cluster one loop, a highly polymorphic region of the AMA1 region that is the target of antibody-mediated inhibitory antibodies, were substituted with alanine. Contrary to the initial hypothesis, the alanine-mutated protein failed to enhance cross-reactive inhibitory activities [46]. A subsequent study used a more conservative approach by mutating only five key polymorphic residues in AMA1 [47]. This approach broadened reactivity across heterologous alleles, but at a cost of reduced overall invasion-inhibitory activity.

In a follow-up study, a multivalent AMA1 vaccine was designed based on four strains (Quadvax) that are broadly representative of most laboratory strains [44]. In *in vitro* assays, Quadvax showed potent inhibition against the four homologous strains in addition to 22 non-vaccine laboratory and recent field isolates [44]. Most importantly, this study showed that mixing only a limited number of diverse strains can dilute the polymorphisms and divert the immunogenicity toward conserved regions of the AMA1 proteins. These findings suggest that it is possible to cover antigen diversity by carefully selecting a subset of diverse strains. In a similar approach but using sequences generated from field samples and a Bayesian clustering algorithm that separated alleles into groups based on genetic distance, it was possible to reduce the number of strains relevant for an AMA1-based vaccine to six [15]. A recent study suggested that a pentavalent AMA1 vaccine may induce protection against a very large number of malaria parasites worldwide [48]. While mixing native allelic proteins has been the most successful way to overcome diversity of pathogens, the cost of manufacturing such a complex multivalent vaccine against malaria may be prohibitive.

It is worth noting that immuno-dampening, which consists of mutating highly polymorphic residues to reduce their immunogenicity [47], may affect the three dimensional structure of the protein resulting in reduced immunogenicity [46] while grouping haplotypes based on a very finite number of sequences may results in clusters that are not representative of the worldwide distribution of variants as observed for AMA1 [9].

#### 3.1.2. Serological approaches

Since sequence differences and similarities may not strongly predict antigenic differences, it can be informative to select alleles or constructs for vaccines based on antigenic properties. This is an established approach for vaccines against viral and bacterial pathogens. For example, antibodies against a panel of reference strains are used to serotype infections in a population. For MSP2, it is probably reasonable to group different polymorphic variants into two major serogroups or serotypes, which represent the two allelic families. However, in the case of AMA1, classifying different variants into serogroups is more challenging. Importantly, emerging data suggest that antigenic diversity of AMA1 is more restricted than would be expected from sequence analyses [18]. Analyses of the specificity and cross-reactivity of functional anti-AMA1 antibodies generated by immunization of experimental animals against different isolates suggest that a vaccine comprised of three to five different alleles may be sufficient to inhibit all isolates [18,39,40,48]. Complementary studies of naturally-acquired antibodies using multi-antigen competition ELISAs also suggested that the inclusion of three or four different AMA1 alleles in a vaccine may be sufficient to cover antigenic diversity, provided the correct alleles are selected [17]. This approach may be helpful in selecting alleles for inclusion in a multi-allele vaccine. A prominent feature of naturally-acquired antibodies to AMA1 is that they target highly polymorphic inhibitory epitopes [49]; therefore developing strategies to refocus antibodies toward conserved or less polymorphic epitopes should reduce the propensity for immune evasion.

#### 3.1.3. Molecular epidemiology techniques

The use of field data to select variants to include in multivalent vaccines was pioneered by researchers working on influenza and pneumococcal vaccines. From a septavalent vaccine in 2000, the number of serotypes in the pneumococcal conjugate vaccine was increased to 13 in 2010 to cover the most relevant serotypes identified in the field [50]. The pneumococcal polysaccharide vaccine covers 23 of the 90 capsular serotypes observed worldwide [51]. A similar approach is being used by malaria vaccine developers in attempts to circumvent the genetic diversity of vaccine antigens and improve the overall efficacy of malaria vaccine candidates [52,53]. These findings in addition to antigenic data were used to infer which haplotypes could be included in cross-protective multivalent vaccines and to guide assessment of allele-specific efficacy in field trials of two AMA1 vaccines [7,10,54].

Polymorphisms responsible for immune escape may be far apart on the linear sequence but cluster in three-dimensional space (*i.e*. conformational epitopes) and therefore the definition of antigenic diversity from genetic data is not straightforward. Mapping polymorphisms to three-dimensional protein structures can identify clustered residues that may form conformational epitopes [8]. Molecular epidemiological studies that track the dynamics of polymorphism in natural populations and during vaccine trials, and cross-sectional surveys which use population genetics to define regions under selection and assess the frequency of different polymorphisms and haplotypes, have been used to pinpoint the specific polymorphic amino acids that mediate immune escape [11,9,12]. Since alleles under balancing selection tend to maintain moderate allele frequencies within and between populations, strategies including focusing only on polymorphisms present in a substantial proportion of isolates or those with similar frequencies between populations have been used to predict important polymorphisms [55,56]. Although these approaches have their limitations, they can help to narrow the focus on specific regions or polymorphism. Combined with serological approaches these methods can help to predict key immune escape polymorphisms. If done as part of the development of novel antigens as vaccine candidates, this would help to define different serotypes and thus advance the selection of appropriate alleles for inclusion in a diversity-covering vaccine [16].

Experimental data suggesting that cross-protection against diverse strains can be achieved [57] are encouraging, but *in vitro* data do not necessarily predict cross-protective immune responses in vaccinated humans. Antigen diversity is one reason why *in vitro* or animal results do not readily translate into efficacious vaccines in humans. An MSP1 vaccine that gave rise to human antibodies that inhibited growth of three diverse *P. falciparum* isolates *in vitro* [42] turned out not to protect against clinical malaria caused by diverse parasites in the field [6]. New subunit malaria vaccines should therefore be designed based on comprehensive information about antigenic diversity in natural parasite populations (Fig. 2). While it might be feasible to design efficacious multivalent vaccines that target proteins with a manageable number of antigenically distinct forms, the notion of developing a broadly efficacious vaccine targeting an extremely polymorphic antigen may be daunting. In addition, there may also be vaccine space limits for the total antigen dose in a vaccine formulation.

#### 3.1.4. Conserved target approaches

An ideal malaria vaccine might be based on conserved antigens that induced broadly efficacious antibodies. Conserved antigens are often assumed to be less immunogenic than the highly polymorphic antigens used in first generation malaria vaccine candidates, but malaria incidence studies and clinical trials suggest that conserved malaria antigens can induce strong and specific immune responses in animals [58] and humans [59]. One approach used to reduce the effect of antigen diversity on vaccine efficacy consists of expressing fragments of vaccine antigens that are target of conserved monoclonal antibodies. The challenge now is to identify the three dimensional structure of relevant antigens to design and express fragments that can structurally mimic the conserved mAb epitopes [60,61]. Peptides/proteins that are generated can be tested by ELISA and/or flow cytometry using sera collected in endemic regions. Fragments that are antigenic and/or cross-reactive can be prioritized as malaria vaccine candidates. This strategy was applied to identify a region of the gene encoding RH5 that is the target of inhibitory antibodies [62]. Immunization of mice with virus-like particles displaying the short sequence induced antibodies that had strong invasion inhibitory activities [44]. It remains to be seen how this epitope display approach for Rh5 would compare to the full-length vaccine. However, vaccines based on epitope display approaches in HIV have thus far failed to recapitulate the inhibitory activity of the parent mAb [63]. A recent non-human primate trial of a RH5-based vaccine provides evidence that a protein with limited polymorphism can induce protection against heterologous parasites [58].

The short fragment approach could be limited by the conformation of fragments that are being expressed. Small peptides or proteins fragments are expressed from synthetic genes that are mostly linear. Therefore, the disulfide bonds that link amino acids that are not contiguous may be missing resulting in proteins that may not retain important structural features. Such proteins in a vaccine formulation may not be efficacious due to a lack of immunogenicity or the production of antibodies that are not specific to it targeted malaria antigen.

## 4. Whole proteome and parasite methods

### 4.1. Protein microarrays

A protein microarray technology first used to measure seroreactivity to thousands of proteins derived from the *P. falciparum* reference genome [64] has been adapted to evaluate allele-specific humoral immunity by printing dozens to hundreds of variants of antigens of interest on the array [27,65]. This diversity array was probed with sera from adult and children from malaria transmission areas during consecutive malaria infections and/or clinical episodes to assess the dynamics of malaria immune responses. This new tool holds potential to help identify variants that are immunogenic, as well as those that are cross protective. The variants identified through this process could be used as components of a multivalent sub-unit malaria vaccine.

Another new approach is offered by an ultra-dense array comprised of short oligomers with near complete amino acid overlap of all antigens printed on the array, with the capacity to screen sera against 2.1 million peptides, covering essentially entire microbial proteomes [66]. In addition to evaluating antigen immunogenicity, the technique identifies residues that are antibody binding sites. A possible limitation of the peptide arrays is that short linear peptides may not emulate the native protein structure, resulting in non-specific binding of antibodies. Other approaches using more targeted arrays of purified and validated proteins, often full length, have identified new lead antigens that could be considered in vaccine development [67,68].

### 4.2. Whole parasite approaches

The successful immunization of birds and monkeys with killed whole malaria parasites in the early 20th century [69–72] was followed in the mid-1970s by experimental challenge using mosquitoes to immunize humans with metabolically active, radiation-attenuated sporozoites [73,74]. Although sterile protection against challenge was achieved, the concept was judged impractical until recently when advances in parasite cultivation methods and mosquito breeding have resulted in the development of a whole sporozoite malaria vaccine [75]. Recently, in a Controlled Human Malaria Infection (CHMI) study to test the efficacy of a whole irradiated sporozoite malaria vaccine, subjects receiving high doses of the vaccine were fully protected against subsequent homologous experimental malaria infection [32]. This success has raised hopes for a highly efficacious malaria vaccine in the near future. Very limited data from human heterologous challenge studies provide some evidence for both cross-strain and even cross-species protection [76]. However, the possibility that diversity-covering approaches such as a multi-strain vaccine may be required for whole organism vaccines is supported by a recent *in vitro* study of the allele-specific efficacy of *Plasmodium yoelii yoelii*. Under a controlled-infection and treatment procedure, immunity to pre-erythrocyte antigens was specific to the strains used during infection [77]. An identical study [78] using *Plasmodium chabaudi* sporozoites likewise found that the immunity against the pre-erythrocytic stage of the parasite life cycle was allele-specific.

Ongoing or planned phase 2 trials in endemic settings in Mali, Tanzania and Burkina Faso will ascertain whether this whole organism vaccine provides strain-transcending immunity. These field trials will also provide an opportunity to perform a genome-wide sieve analysis to identify genetic loci under selection by the vaccine, although larger studies may be required to achieve the sample size necessary to detect evidence of selection in genome-wide sieve analyses. Such analyses may provide invaluable information not only about how to select strains for a broadly efficacious multi-strain whole organism vaccine, but also about what specific polymorphic antigens contribute to allele-specific immunity to P. *falciparum*.

## 5. Discussion and perspectives

Pathogen populations characterized by polymorphic surface antigens appear to have evolved in response to immune selection by the human host by expressing polymorphic surface antigens that retain function despite significant cross-strain variation [79]. A leading method used by vaccine developers for other microorganisms is to cover antigenic diversity by selecting clinically relevant strains that are representative of the distribution of the microorganism in their vaccine design. Salk [37] methodically classified circulating polio strains prior to the selection of one the three strains that he would use in the inactivated polio vaccine. Moreover, the conjugate 13-valent and the 23-valent polysaccharide pneumococcal vaccines have been designed to overcome the genetic diversity of 90 serotypes that are distributed worldwide. However, malaria parasites are much more complex with scores of surface proteins with extensive diversity that is not well understood. Furthermore, commonly used reference strains are quite often found infrequently in naturally circulating parasite populations. The “freezer epidemiology” approach of selecting available and convenient strains for the design of a malaria vaccine has therefore shown it limitations. The design of a broadly efficacious malaria vaccine should take into account the baseline genetic and antigenic diversity (Fig. 1) of candidate antigens [10,16,12]. This approach (reverse vaccinology) [80], which uses a combination of bioinformatics analyses to select antigens and molecular epidemiology to select the most prevalent allele/strain in the field, offers a promising approach for designing a broadly efficacious malaria vaccine. In addition to all these approaches, the design of chimeric proteins is a strategy that is being used to improve malaria vaccine efficacy [81].

## 6. Concluding remarks

Almost all first generation malaria vaccines were designed without consideration of antigen variant prevalences in the field. Molecular epidemiology, genomic epidemiology, serological, and conserved peptide approaches in addition to immuno-dampening have each been used to guide the selection of relevant variants. These approaches are all limited by relying essentially on the linear sequences for analysis. Moreover, while it may be feasible to design multivalent vaccines based on few variants, designing vaccines that include more than 10 variants will require tremendous technical advances. However, a multivalent malaria vaccine may offer redundancies that compensate for failure of individual component antigens to induce functional antibodies. Whole-organism malaria vaccines, whether based on a single strain or multiple strains, may overcome antigenic diversity by offering even greater redundancy.

## Figures and Tables

**Table 1:** Malaria vaccines and allele-specific efficacy.

Vaccine	Antigen	Stage	Allele-specific efficacy	Reference
RTS,S	Circumsporozoite protein (CSP)	Pre-erythrocytic	No	[30]
AMA1-C1	Apical membrane antigen 1 (AMA1)	Erythrocytic	No	[52]
FMP2.1/AS01	Apical membrane antigen 1 (AMA1)	Erythrocytic	Yes	[7]
Combination B	Merozoite surface protein 2 (MSP2), MSP1 Ring-infected erythrocyte surface antigen (RESA)	Erythrocytic	Yes	[4]
SPf66	Merozoite surface protein 2 (MSP1)	Erythrocytic	Yes	[33]
